# Excitability Tuning of Axons by Afterdepolarization

**DOI:** 10.3389/fncel.2019.00407

**Published:** 2019-09-06

**Authors:** Haruyuki Kamiya

**Affiliations:** Department of Neurobiology, Hokkaido University Graduate School of Medicine, Sapporo, Japan

**Keywords:** axon, action potential, afterdepolarization, propagation, short-term plasticity

## Abstract

The axon provides a sole output of the neuron which propagates action potentials reliably to the axon terminal and transmits neuronal information to the postsynaptic neuron across the synapse. A classical view of neuronal signaling is based on these two processes, namely binary (all or none) signaling along the axon and graded (tunable) signaling at the synapse. Recent studies, however, have revealed that the excitability of the axon is subject to dynamic tuning for a short period after axonal action potentials. This was first described as post-spike hyperexcitability, as measured by the changes in stimulus threshold for a short period after an action potential. Later on, direct recordings from central nervous system (CNS) axons or axon terminals using subcellular patch-clamp recording showed that axonal spikes are often followed by afterdepolarization (ADP) lasting for several tens of milliseconds and has been suggested to mediate post-spike hyperexcitability. In this review article, I focused on the mechanisms as well as the functional significance of ADP in fine-scale modulation of axonal spike signaling in the CNS, with special reference to hippocampal mossy fibers, one of the best-studied CNS axons. As a common basic mechanism underlying axonal ADP, passive propagation by the capacitive discharge of the axonal membrane as well as voltage-dependent K^+^ conductance underlies the generation of ADP. Small but prolonged axonal ADP lasting for several tens of milliseconds may influence the subsequent action potential and transmitter release from the axon terminals. Both duration and amplitude of axonal spike are subject to such modulation by preceding action potential-ADP sequence, deviating from the conventional assumption of digital nature of axonal spike signaling. Impact on the transmitter release is also discussed in the context of axonal spike plasticity. Axonal spike is subject to dynamic control on a fine-scale and thereby contributes to the short-term plasticity at the synapse.

## Dynamic Tuning of Axon Excitability by ADP

**FIGURE 1:**
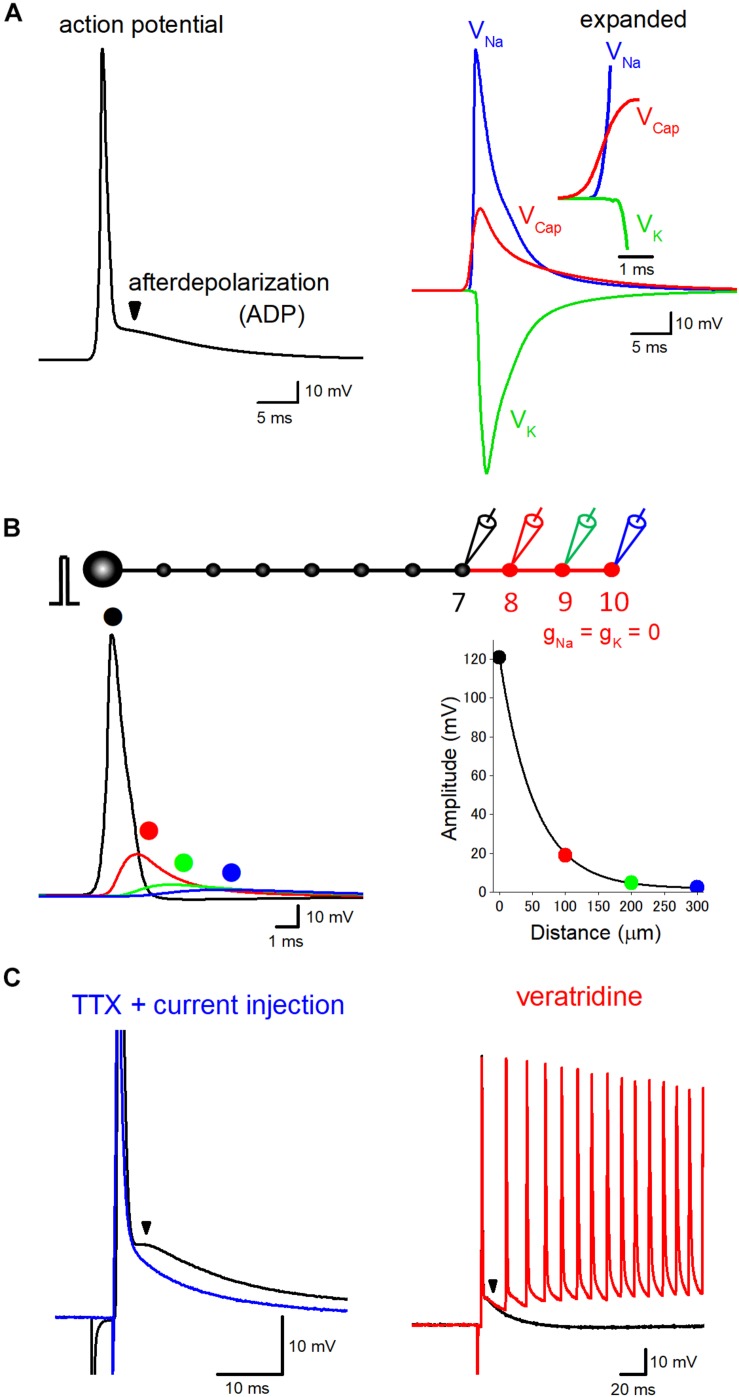
Components of axonal ADP. **(A)** Basic components constituting axonal action potential-ADP sequence. Action potentials recorded from axon or the terminal are typically followed by prolonged ADP lasting for several tens of milliseconds. The left trace represents the time course of ADP in the hippocampal mossy fiber model calculated at negative membrane potentials of –100 mV. The right traces shows ionic components due to activation of voltage-dependent Na^+^ (V_Na_, *blue*) and K^+^ conductance (V_K_, *green*), the electrical component due to capacitive discharge (V_Cap_, *red*) also substantially contribute to the prolonged ADP. V_Na_, V_K_, and V_Cap_ were calculated by subtraction of membrane potentials calculated by removal of Na^+^ and/or K^+^ conductance from the terminals. Sum of V_Na_, V_K_, and V_Cap_, therefore, was identical with action potential-ADP sequence shown in the left trace. The inset represents the time-expanded traces showing timing and sequence of the onset of V_Na_, V_K_, and V_Cap_. The electrical component (V_Cap_) precedes the ionic components (V_Na_ and V_K_) to trigger action potential at the downstream axons. **(B)** Passive propagation of upstream action potential. Using a model simulation of *en passant* axon (10 boutons spaced every 100 μm) mimicking the structure of hippocampal mossy fibers, the relative contribution of passive propagation to the downstream ADP was evaluated by removing voltage-dependent ionic conductance (g_Na_ and g_K_) from 8th–10th boutons and axon, as shown in *red*. Brief current injection into the soma elicited action potential which propagates faithfully to the 7th bouton without attenuation. The amplitude of depolarization decreased successively from the 8th bouton, and the time course was slowed down along the distance due to filtering by axon cable. The decay of the peak amplitude along the distance was fitted by a single exponential curve with 53 μm for the distance with a reduction to 1/e (37%). **(C)** Boosting axonal ADP by slow Na^+^ channels. In direct whole-cell recording experiment from hippocampal mossy fiber terminals, ADP is partly mediated by tetrodotoxin (TTX)-sensitive slow activating Na^+^ channels. In the presence of TTX, action potentials and ADP were abolished, but brief current pulse injection restored sharp depolarization followed by slow relaxation (*blue*). Veratridine, an inhibitor of inactivation of Na^+^ channels, enhanced the ADP and sometimes overlaid by multiple spiking elicited by a single stimulus in mossy fiber axons (*red*). Modified with permission from Ohura and Kamiya (2018b) and Kamiya (2019).

The axon carries neuronal information as a form of action potentials which reliably propagate for a long distance without attenuation (Debanne et al., 2011). A regenerative nature of spike generation provides digital property beneficial to reliable and ultrafast axonal signaling in the nervous system (Bean, 2007). Recent studies however, updated the classical view of digital axonal signaling to impart analog modification by the preceding neuronal activity (Zbili and Debanne, 2019). Such a use-dependent analog modification of axonal spike is possibly due to dynamic control of excitability of axon by the preceding neuronal activity for a short period up to tens to hundreds of milliseconds after generation of action potential (Gardner-Medwin, 1972; Zucker, 1974; Bucher and Goaillard, 2011). This post-stimulus change in the excitability of axon was mediated by ADP following action potential (Figure 1A), which may be important for temporal integration of axonal excitability and short-term plasticity of presynaptic transmitter release (Barrett and Barrett, 1982; Ohura and Kamiya, 2016). In this review, I focused on the recent progress in understanding the mechanisms as well as the functional significance of axonal afterdepolarization (ADP) in paired-pulse modulation and short-term synaptic plasticity.

## Components of Axonal ADP

ADP following action potentials is common process observed in the nervous systems in both vertebrate and invertebrate axons (Barrett and Barrett, 1982; Borst et al., 1995; Geiger and Jonas, 2000). A small but prolonged depolarization during ADP may important factor for activity tuning of axonal excitability as well as presynaptic transmitter release. Figure 1A shows the typical time course of ADP in hippocampal mossy fiber model (Engel and Jonas, 2005) calculated at negative membrane potentials (−100 mV) which shows similar time course with those recorded from mossy fiber boutons experimentally (Geiger and Jonas, 2000; Ohura and Kamiya, 2018b). This model assumes a Hodgkin Huxley-type gating adapted to channels those recorded in mossy fiber terminals and implemented with K^+^ channel inactivation. There is a considerable number of studies addressing the mechanisms underlying ADP, and it seems to be reasonable to comprehend that axonal ADP consists of common basic mechanisms and of additional specific mechanisms to particular axons.

As a common mechanism underlying axonal ADP, passive propagation of upstream action potential via axon cable has been shown to consist of a basic component of ADP (Barrett and Barrett, 1982; Borst et al., 1995; David et al., 1995). Additional contribution of several specific mechanisms, e.g., activation of slow sodium current like resurgent or persistent sodium current (Kim et al., 2010; Ohura and Kamiya, 2018b), accumulation of potassium ions surrounding axons (Malenka et al., 1981; Kocsis et al., 1983; Meeks and Mennerick, 2004), or activation of autoreceptors of glutamate and GABA (Kamiya et al., 2002; Ruiz et al., 2003; Stell et al., 2007; Zorrilla de San Martin et al., 2017), have been also suggested.

## Passive Propagation Via Axon Cable

Cable property of axon confers a delayed depolarization due to capacitive discharge from the upstream action potential. Such a passive propagating component consists of axonal ADP at least in part. Consistent with this notion, ADP recorded from a calyx of Held axon terminals was shown to be unaffected by tetrodotoxin focally applied to the axon terminals (Borst et al., 1995), suggesting the possible axonal origin of ADP. Passive nature of ADP has also supported the finding that ADP and passive electrotonic response to step hyperpolarizing current injection showed similar time courses in motor axons (Barrett and Barrett, 1982) or in hippocampal mossy fiber axons (Ohura and Kamiya, 2018b).

To evaluate quantitative contribution of passive propagation in action potential-ADP sequence, time courses of the ionic components due to activation of voltage-dependent Na^+^ and K^+^ channels (V_Na_ and V_K_) as well as electrical components due to capacitive discharge (V_Cap_) are compared (Figure 1A) in the hippocampal mossy fiber model used in our recent simulation study (Kamiya, 2019). The amplitude of V_Cap_ is more than one-third of V_Na_, suggesting a substantial contribution of passive electrical components in ADP, in agreement with the experiment of brief current injection in the presence of tetrodotoxin, a blocker of voltage-dependent Na^+^ channels (Ohura and Kamiya, 2018b). The onset of V_Cap_ preceded V_Na_ to trigger action potentials as shown in the inset with an expanded time scale.

It should be noted that passive propagation may distribute over a relatively long distance. The length constant of the axon was estimated as 455 μm in layer V pyramidal neurons in ferret cortex (Shu et al., 2006), 450 μm in rat hippocampal granule cell (Alle and Geiger, 2006), and 121 μm in cultured rat Purkinje cell (Zorrilla de San Martin et al., 2017). Although the length constant evaluated by long current pulse injection was estimated as 171 μm in our model of hippocampal mossy fibers (Kamiya, 2019), action potentials decline with shorter space constant of 53 μm for decay to 1/e (Figure 1B) possibly reflecting steeper filtering of fast voltage transient during action potentials. Passive propagation filtered by axon cable may thereby substantially impact the time course of action potential-ADP sequence recorded from the downstream axon.

It is worth considering whether subthreshold fluctuation of somatic membrane potentials occurring *in vivo* may also passively propagate to the axon. Long-range propagation of somatic depolarization into the axon has been demonstrated for hippocampal mossy fibers (Alle and Geiger, 2006) and for cortical pyramidal cell axons (Shu et al., 2006). In both studies, axonal depolarization enhanced transmitter release from the axon terminals, while the effect was limited to the proximal portion of the axons (see also Scott et al., 2008) due to passive attenuation of depolarization with the distance from the soma. On the contrary, ADP following action potentials is expected to equally distribute along the course of the axon, since action potentials propagate without attenuation. Therefore passive propagating components of axonal ADP is not depending on the distance from the soma and differed from the subthreshold somatic depolarization in this point.

Contribution of passive propagation is important for transmitter release not only from *en passant* boutons like the hippocampal mossy fibers, but also from bouton terminaux like calyx of Held, since it has been shown that Na^+^ channels are excluded from the terminals but are expressed only in the axonal heminode in the distal axon (Leão et al., 2005). The depolarization at the active zone, which is directly related to Ca^2+^ entry responsible for transmitter release, is therefore mostly reflecting passive propagation from the heminode region to the terminal boutons.

## Voltage-Dependency of Axonal ADP

Quite puzzling observations are ADP recorded from axons or the terminals showed clear voltage-dependency. The size of ADP decreased upon depolarization of the initial membrane potentials (Begum et al., 2016; Sierksma and Borst, 2017; Ohura and Kamiya, 2018b), and sometimes reversed in polarity at more positive membrane potentials. It was also demonstrated that hyperpolarizing current injection decreased ADP (Kim et al., 2010). These findings are difficult to interpret with the passive nature of capacitive discharge of the axonal membrane, which is fundamentally voltage-independent. Therefore, the additional contribution of voltage-gated conductance which provides voltage-dependency to axonal ADP (Kamiya, 2019) must be taken into consideration.

## K^+^ Channels Shape the Initial Phase of Axonal ADP

As additional conductance that potentially confers voltage-dependency to axonal ADP, the contribution of voltage-dependent K^+^ conductance, which predominantly mediates fast repolarization of action potential (Storm, 1987; Dodson et al., 2003; Wissmann et al., 2003), was suggested. The time course of K^+^ conductance somewhat outlasts the duration of action potentials and necessarily contributes to the subsequent ADP time course. Consistent with this notion, voltage-dependent K^+^ conductance shapes a characteristic breakpoint at the initial phase of axonal ADP, possibly due to superimposed hyperpolarizing K^+^ conductance on the passive depolarizing component. Sum of voltage-independent passive component and voltage-dependent K^+^ channel component with the various combination in hippocampal mossy fiber model nicely reconstruct the characteristic time course as well as typical voltage-dependency of action potential-ADP sequence (Kamiya, 2019). It seems to be reasonable to speculate that various reported values of apparent reversal potential of axonal ADP (Begum et al., 2016; Sierksma and Borst, 2017; Ohura and Kamiya, 2018b) reflect the various contribution of passive propagation and voltage-dependent K^+^-channel components.

## Slow Na^+^ Current Boosts ADP in Some Axons

In addition to the common basic mechanisms of passive propagation and voltage-dependent K^+^ conductance, the contribution of slow Na^+^ current at a certain type of axon has been suggested. ADP recorded from soma sometimes are mediated by slow voltage-dependent Na^+^ currents such as persistent-type I_NaP_ or resurgent-type I_NaR_ (Raman and Bean, 1997; Yue and Yaari, 2004; Yue et al., 2005; D’Ascenzo et al., 2009). Using direct recording from the calyx of Held axon terminals, thereafter, it has been shown that the resurgent-type Na^+^ current I_NaR_ shapes slow time course of axonal ADP (Kim et al., 2010). In contrast, it was reported that ADP was unaffected by local application of tetrodotoxin surrounding the recorded terminals at the same axon terminals (Borst et al., 1995), although the reason of the different results in these studies is unclear. This discrepancy may be explained by the finding that Na^+^ channels are not located in the calyx terminal but in the distal heminode region of the axon (Leão et al., 2005). Recently we also have reported that ADP at hippocampal mossy fiber terminals is also partially mediated by voltage-dependent Na^+^ current (Ohura and Kamiya, 2018b) since it was suppressed by a Na^+^ channel blocker tetrodotoxin and was enhanced by an inhibitor of inactivation of Na^+^ channel veratridine (Figure 1C), as shown for ADP at the calyx of Held terminals (Kim et al., 2010). These slow Na^+^ channels raise axonal excitability for a while and help to support faithful spiking of axons during repetitive stimuli. Their molecular identity, as well as the precise subcellular localization of the axonal slow Na^+^ channels, remain to be determined. It is also uncertain whether this mechanism is generally applicable to other types of axons in different brain regions.

## Accumulation of K^+^ Surrounding Axons

It was reported that stimulation of parallel fiber axons in rat cerebellum cause elevation of extracellular K^+^ concentration surrounding axons and caused prolonged depolarization and hyperexcitable period lasting for a hundred of milliseconds (Malenka et al., 1981; Kocsis et al., 1983). It is intriguing to speculate that action potential in axon or EPSPs in postsynaptic neurons caused elevation of extracellular K^+^ concentration in the extracellular space and axon to depolarize by elevated K^+^ concentration surrounding axons. It will be worth testing the possible contribution of astroglia in shaping axonal ADP since local extracellular K^+^ buffering depends mainly on astroglia. It is also intriguing to test the roles of gliotransmitters in shaping axonal ADP, since glutamate released from astroglia was shown to broaden action potentials locally (Sasaki et al., 2011). However, the increase in elevated levels of K^+^ was observed only when strong and repetitive stimuli were given. The single shock-induced K^+^ increase was detectable only when a K^+^ channel blocker 4-AP was applied. Therefore, it seems to be that this mechanism may not contribute to the generation of ADP physiologically by single action potential at single axon.

## Contribution of Autoreceptor Activation in Axonal ADP

In some specific axons, it has been shown that activation of autoreceptors of GABA (Pouzat and Marty, 1999; Zorrilla de San Martin et al., 2017) participates in axonal ADP. Activation of axonal GABA_A_-autoreceptors at cerebellar interneuron axons causes excitatory GABAergic autoreceptor currents (Pouzat and Marty, 1999), possibly due to higher chloride concentration in axoplasm, and facilitate transmitter release and increase neuronal firing rate (Mejia-Gervacio and Marty, 2006). Similar depolarizing autoreceptor current was also reported for cultured Purkinje cell axons (Zorrilla de San Martin et al., 2017). It was also suggested that kainate-type glutamate receptors may assist ADP in hippocampal mossy fiber axons recorded optically using voltage-sensitive dye (Kamiya et al., 2002). It should be noted that autoreceptor activation surely contributes to a certain subset of axons in the nervous system, this is not a common mechanism for all types of the axon, but a relatively rare and specific contribution to axonal ADP.

## Impact of Axonal ADP on Subsequent Action Potential and Transmitter Release

**FIGURE 2:**
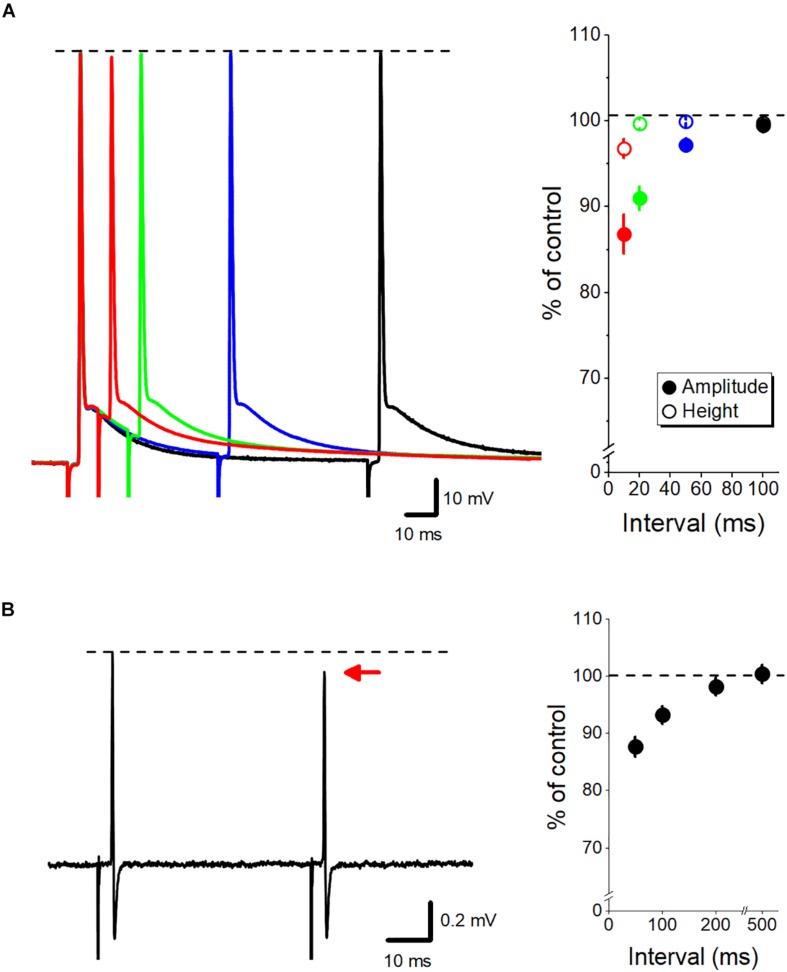
Dynamic fine-tuning of the axonal action potential by ADP. **(A)** Modulation of the subsequent action potential by axonal ADP. Superimposed traces of paired-pulse responses recorded by whole-cell recordings from mossy fiber terminals at 10- (*red*), 20- (*green*), 50- (*blue*), and 100-ms (*black*) intervals. The peak heights of action potentials were almost unaffected (open circles), although the amplitude from the onset of the second action potential (closed circles) was reduced by the paired stimuli at short intervals. **(B)** Paired-pulse depression of axonal spikes recorded from single mossy fiber boutons by loose-patch clamp recordings. The amplitude of the second spike was slightly reduced than the first spike at short intervals. Modified with permission from Ohura and Kamiya (2018a, b).

Slow time course of axonal ADP implies that the excitability of the axon is modulated cumulatively during repetitive stimuli at short intervals. The small and slow depolarization of ADP may affect the states of the voltage-dependent Na^+^ and K^+^ channels and thereby potentially modify the subsequent action potentials. Using whole-bouton recording from mossy fiber terminals, we recently have reported (Ohura and Kamiya, 2018b) that the peak height of the action potentials was almost unchanged, although the amplitudes of the action potentials measured from the elevated initial membrane potential were reduced in paired-pulse stimuli at short intervals (Figure 2A). Elevated membrane potentials by ADP may facilitate steady-state inactivation of voltage-dependent Na^+^ channels (Engel and Jonas, 2005; Rama et al., 2015), and thereby may slow the rising phase of action potentials. Consistent with this, axonal spike recorded extracellularly from single mossy fiber terminal, which is expected to reflect the first derivative of action potential recorded intracellularly (Meeks et al., 2005), displayed short-term depression of the amplitude at short intervals (Ohura and Kamiya, 2018a) as shown in Figure 2B. Inactivation of Na^+^ channels was also suggested for mediating adaptive broadening of spike initiation site during somatic depolarization, as demonstrated by simultaneous soma and axon recordings from hippocampal mossy fibers (Scott et al., 2014). It was also demonstrated that the preceding ADP also enhanced inactivation of K^+^ channels, leading to a use-dependent broadening of action potentials (Geiger and Jonas, 2000; Kole et al., 2007).

Voltage-dependent Ca^2+^ channels are also affected by the preceding ADP. Modulation of presynaptic Ca^2+^ current would be expected to modulate transmitter release and short-term synaptic plasticity (Bischofberger et al., 2002; Li et al., 2007; Zorrilla de San Martin et al., 2017). Although the detailed biophysical mechanism remains to be clarified, facilitation of Ca^2+^ current consequently enhances the subsequent transmitter release from the axon terminals at the culture Purkinje cell axon terminals (Zorrilla de San Martin et al., 2017) and hippocampal mossy fiber terminals (Ohura and Kamiya, 2018b). Impacts on the downstream synaptic transmission is a small but non-negligible role of axonal ADP since the amount of transmitter release is steeply dependent on the amount of Ca^2+^ entry during an action potential supra-linearly (Zucker and Regehr, 2002). In fact, it has been shown that subthreshold depolarization of the calyx of Held raised the Ca^2+^ levels by weak activation of P/Q-type Ca^2+^ channels and enhanced transmitter release from the terminals (Awatramani et al., 2005).

## Conclusion

Recent studies using the direct electrophysiological recordings from axons or axon terminals in the central nervous system have revealed that the excitability of axons is regulated more dynamically than previously thought. In this review article, advances in the understanding of the mechanisms and the functional significance of ADP following axonal action potentials were summarized. It has been demonstrated that electrical signals passively propagate for hundreds of micrometers, and consequently, the passive propagating component due to the capacitive discharge of axons substantially contributes to the propagating action potential-ADP sequence. The changes in the excitability by axonal ADP last for tens or hundreds of milliseconds and therefore may play an important role in temporal integration of neuronal activity and short-term synaptic plasticity. Such a fine-scale dynamics of axonal excitability play pivotal roles in fine-tuning and temporal integration of neuronal network functions.

## Author Contributions

HK conceptualized and designed the study and drafted the manuscript.

## Conflict of Interest Statement

The author declares that the research was conducted in the absence of any commercial or financial relationships that could be construed as a potential conflict of interest.
